# GC-MS/MS Quantification of EGFR Inhibitors, *β*-Sitosterol, Betulinic Acid, (+) Eriodictyol, (+) Epipinoresinol, and Secoisolariciresinol, in Crude Extract and Ethyl Acetate Fraction of *Thonningia sanguinea*

**DOI:** 10.3390/molecules27134109

**Published:** 2022-06-26

**Authors:** Sameh S. Elhady, Elsayed A. Ibrahim, Marwa S. Goda, Mohamed S. Nafie, Hanan Samir, Reem M. Diri, Abdulrahman M. Alahdal, Ama Kyeraa Thomford, Alaa El Gindy, Ghada M. Hadad, Jihan M. Badr, Reda F. A. Abdelhameed

**Affiliations:** 1 Department of Natural Products, Faculty of Pharmacy, King Abdulaziz University, Jeddah 21589, Saudi Arabia; ssahmed@kau.edu.sa; 2 Department of Pharmaceutical Analytical Chemistry, Faculty of Pharmacy, Suez Canal University, Ismailia 41522, Egypt; elsayed_ibrahim@pharm.suez.edu.eg (E.A.I.); hanan.samir@pharm.suez.edu.eg (H.S.); alaaeldeen_mohamed@pharm.suez.edu.eg (A.E.G.); 3 Department of Pharmacognosy, Faculty of Pharmacy, Suez Canal University, Ismailia 41522, Egypt; marwa_saeed@pharm.suez.edu.eg (M.S.G.); gehan_ibrahim@pharm.suez.edu.eg (J.M.B.); 4 Department of Chemistry, Faculty of Science, Suez Canal University, Ismailia 41522, Egypt; mohamed_nafie@science.suez.edu.eg; 5 Medical Administration, Students Hospital, Zagazig University, Zagazig 44519, Egypt; 6 Department of Pharmacy Practice, Faculty of Pharmacy, King Abdulaziz University, Jeddah 21589, Saudi Arabia; rdiri@kau.edu.sa (R.M.D.); amalahdal@kau.edu.sa (A.M.A.); 7 Department of Biomedical Sciences, School of Allied Health Sciences, University of Cape Coast, Cape Coast 03321, Ghana; ama.thomford@ucc.edu.gh; 8 Department of Pharmacognosy, Faculty of Pharmacy, Galala University, New Galala 43713, Egypt; reda.fouad@gu.edu.eg

**Keywords:** *Thonningia sanguinea*, GC-MS/MS, eriodictyol, secoisolariciresinol, EGFR

## Abstract

Medicinal plants are widely used in folk medicine to treat various diseases. *Thonningia sanguinea* Vahl is widespread in African traditional medicine, and exhibits antioxidant, antibacterial, antiviral, and anticancer activities. *T. sanguinea* is a source of phytomedicinal agents that have previously been isolated and structurally elucidated. Herein, gas chromatography combined with tandem mass spectrometry (GC-MS/MS) was used to quantify epipinoresinol, *β*-sitosterol, eriodictyol, betulinic acid, and secoisolariciresinol contents in the methanolic crude extract and its ethyl acetate fraction for the first time. The ethyl acetate fraction was rich in epipinoresinol, eriodictyol, and secoisolariciresinol at concentrations of 2.3, 3.9, and 2.4 mg/g of dry extract, respectively. The binding interactions of these compounds with the epidermal growth factor receptor (EGFR) were computed using a molecular docking study. The results revealed that the highest binding affinities for the EGFR signaling pathway were attributed to eriodictyol and secoisolariciresinol, with good binding energies of −19.93 and −16.63 Kcal/mol, respectively. These compounds formed good interactions with the key amino acid Met 769 as the co-crystallized ligand. So, the ethyl acetate fraction of *T. sanguinea* is a promising adjuvant therapy in cancer treatments.

## 1. Introduction

Medicinal plants are a plentiful source of bioactive phytoconstituents that have drawn attention for their use in treatments of several diseases and in the discovery of new therapeutic drugs. The usage of medicinal plants is central to African traditional medicine. *Thonningia sanguinea* Vahl. (Family: Balanophoraceae) is a widely used medicinal herb throughout tropical Africa [1]. It is well-known as ″ground pineapple’’ in English and widely as ″kwaebedwaa’’ in Ghana [2]. *T. sanguinea* is traditionally used as a natural remedy for anal lesions, hemorrhoids, gonorrhea, dysentery, diarrhea, syphilis, fever, bronchial asthma, urinary incontinence, muscle cramps, sore throat, sexual weakness, and numerous skin diseases, and as an anthelmintic [3,4,5,6,7]. Previous biological studies analyzed the significant antimicrobial effect of a crude extract of *T. sanguinea* on *Escherichia coli*, *Klebsiella pneumoniae*, *Salmonella typhi*, *Salmonella enterica, Pseudomonas aeruginosa*, methicillin-resistant *Staphylococcus aureus* (MRSA), *Eimeria tenella* and *Eimeria necatrix* parasites, *Plasmodium berghei* parasite, and fungus *Aspergillus niger* [4,6,7,8,9,10]. Other studies examined the potent antioxidant effects and free radical scavenging activity of the crude extract of *T. sanguinea* that inhibits H_2_O_2_-induced lipid peroxidation in the liver and CCl_4_-induced hepatotoxicity [3]. *T. sanguinea* was found to inhibit aniline hydroxylase non-competitively, suggesting a reduction in biotransformation of AFB1 to toxic metabolites [11]. Due to its health benefits, *T. sanguinea* is commercially available in Ghana under the name of CAMPA-T^®^ and NINGER^®^. Further chemical studies were carried out to isolate and identify the phytoconstituents that contribute to the above-mentioned biological effects. In 2000, both thonningianins A and B, with potent free radical scavenging activity, were isolated [4]. Then, Thomford et al. isolated two glucocerebrosides, seven triterpenes, five lignans, and one flavanone [2]. In addition, ten dihydrochalcone glucoside derivatives, phenylpropanoid coniferin, and lignans were isolated and structurally identified [12]. Recently, chemical profiling of the crude extract of *T. sanguinea* using liquid chromatography combined with tandem mass spectrometry (LC-MS/MS) revealed the presence of a high percentage of phenolic compounds that contribute to the cytotoxic effect of the crude extract [13]. *T. sanguinea* exhibited promising cytotoxic activity against the breast cancer MCF-7 and hepatocellular carcinoma HepG2 cell lines, arresting the cell cycle at the G2/M phase [13]. Some of its previously isolated compounds, namely *β*-sitosterol, betulinic acid, eriodictyol, epipinoresinol, and secoisolariciresinol, were reported for their cytotoxic activity against different cell lines. For example, *β*-sitosterol induced G1 arrest and caused depolarization of the mitochondrial membrane potential in MDA-MB-231 (breast carcinoma cells) [14]. Betulinic acid inhibited proliferation and induced apoptosis in a number of cancer cell lines including breast, colon, brain, prostate, and leukemia [15]. Eriodictyol exhibited significant anticancer activity toward the A549 human lung cancer cell line [16]. Additionally, epipinoresinol showed significant antiproliferative effects when tested against two types of colon cancer cell lines (HCT116 and SW480) [17]. Finally, secoisolariciresinol was shown to act as a re-sensitizer of P-glycoprotein-dependent doxorubicin-resistance NCI/ADR-RES cancer cells [18].

In this study, we developed and validated a fast and sensitive technique using gas chromatography combined with tandem mass spectrometry (GC-MS/MS) for the quantitative analysis of *β*-sitosterol, betulinic acid, eriodictyol, epipinoresinol, and secoisolariciresinol in both the ethyl acetate fraction and crude methanolic extract of *T. sanguinea* for the first time. We also provide a brief illustration of their anticancer activity through a molecular docking study showing their contributions to the cytotoxic effect of the crude extract.

## 2. Results and Discussion

### 2.1. Validation Parameters of Analytical Method Using GC-MS/MS

**Figure 1:**
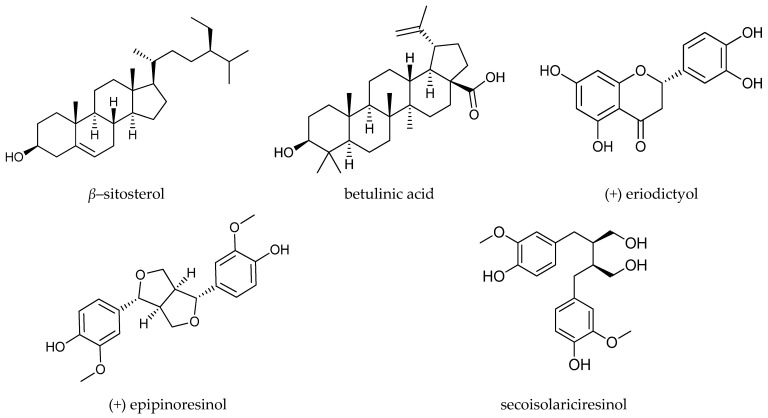
Structures of the analyzed compounds.

Gas chromatography tandem mass spectrometry (GC-MS/MS) is a key technological stage used for secondary metabolite profiling in living organisms. The GC-MS/MS technique can be used to detect a wide range of metabolites from different chemical classes [19,20,21,22]. In this study, the GC-MS/MS analytical method was adopted to facilitate the identification and quantification of *β*-sitosterol, betulinic acid, eriodictyol, epipinoresinol, and secoisolariciresinol (Figure 1).

The proposed analytical method was validated in accordance with the ICH guidelines for accuracy, precision, linearity, detection limit (LOD), quantitation limit (LOQ), specificity, and sensitivity. These validation parameters are described below.

#### 2.1.1. Linearity

The linearity of the proposed GC-MS/MS method was examined by analyzing the standard solution of each studied compound (*β*-sitosterol, betulinic acid, eriodictyol, epipinoresinol, and secoisolariciresinol) in a series of different concentrations under the optimum experimental conditions described in the Materials and Methods section. Seven concentrations were chosen, ranging between 7.0 and 12.0 μg/mL for each compound. The calibration curves of each compound were obtained by plotting the different concentrations of each standard against each corresponding peak area, applied in triplicate. Statistical analysis of the data was carried out using a linear regression analysis. The linear relationship was obtained over the concentration range of 7.0–12.0 μg/mL with a correlation coefficient (R^2^) above 0.99 for all compounds (Table 1).

#### 2.1.2. Limit of Detection and Limit of Quantification

The limit of detection (LOD), based on a signal-to-noise ratio approach, was 0.08, 0.06, 0.08, 0.12, and 0.11 μg/mL for *β*-sitosterol, betulinic acid, eriodictyol, epipinoresinol, and secoisolariciresinol, respectively. The limit of quantitation (LOQ) was 0.26, 0.18, 0.25, 0.4, and 0.38 μg/mL for *β*-sitosterol, betulinic acid, eriodictyol, epipinoresinol, and secoisolariciresinol, respectively (Table 1).

#### 2.1.3. Precision and Accuracy

The intra- and inter-day precisions and accuracy of the five analytes were based on the analysis of three different concentrations of each analyte applied in triplicate, either on the same day for intra-day precision or on different days for inter-day precision (Table 2). Precision, which is indicated by the coefficient of variation (CV%), was less than 9.5%, while accuracy ranged from 98% to 102%. These results showed that the current method had good precision and accuracy.

#### 2.1.4. Specificity and Sensitivity

No endogenous interference was found in the retention times of the analytes. The blank sample had no significant interference caused by any endogenous components in the retention times of the five studied analytes.

### 2.2. Application of Validated Analytical Method

In this study, the GC–MS/MS technique was successively applied to determine the concentrations of *β*-sitosterol, betulinic acid, eriodictyol, epipinoresinol, and secoisolariciresinol in a *Thonningia sanguinea* methanolic extract and its ethyl acetate fraction (Table 3; Figures S1–S5 in Supplementary Data). The concentrations of both *β*-sitosterol and betulinic acid in the methanolic crude extract were higher than those found in the ethyl acetate fraction. This occurred due to the higher affinity of these compounds for non-polar fractions (*n*-hexane fraction) than for moderate or polar fractions (ethyl acetate fraction or *n*- butanol fraction). Eriodictyol, epipinoresinol, and secoisolariciresinol were more concentrated in the ethyl acetate fraction of moderate polarity than in the methanolic crude extract. So, we recommend using the ethyl acetate fraction for future nanoformulation studies of cancer treatments.

These compounds were selected for our study based on their previously reported cytotoxic activity. Recently, herbal products have drawn attention as an adjuvant therapy in treatment of different types of cancer. Eriodictyol exhibited cytotoxic effects against the human A549 lung cell line through the induction of apoptosis and G2/M cell cycle arrest [16]. Secoisolariciresinol diglucoside showed cytotoxic effects against different malignant tumors, including breast, lung, and colon cancers, due to its antiproliferative and antioxidant activities [23]. Epipinoresinol upregulated the tumor suppressor gene P53 that inhibits the proliferation of the HCT116 colon cancer cell line [17]. Additionally, *β*-sitosterol showed a chemo-prophylactic agent in colon carcinogenesis, while betulinic acid was incorporated into chemotherapeutic agents due to its ability to inhibit invasion of lung cancer cells [24,25]. So, it was a strong motive to quantify these phytomedicinal agents in *T. sanguine* herbal drugs, and to interpret their virtual mechanisms of binding toward their respective targets using a molecular docking simulation.

### 2.3. Molecular Docking Studies of Isolated Compounds toward EGFR

**Figure 2:**
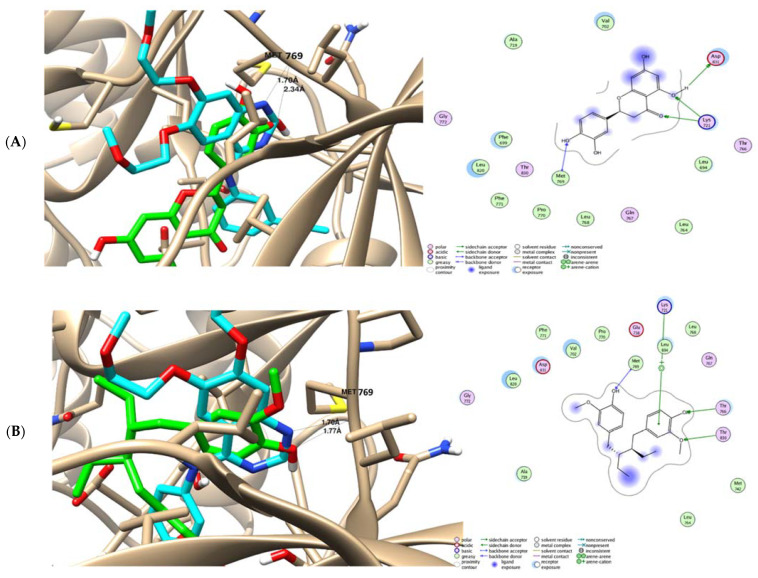
Binding disposition and analysis of ligand–receptor interactions of the two docked compounds inside the EGFR protein: (**A**) (+) eriodictyol and (**B**) secoisolariciresinol, with 2D and 3D interactive images. The co-crystallized ligand is cyan-colored, while the docked compounds are green-colored. The EGFR protein (PDB = 1M17) was freely accessible from the protein data bank.

Molecular docking is an effective, sensitive, and cheap method for drug layout and testing [16]. This method introduces a molecule onto the binding spot of the macromolecule in a non-oval fashion, ensuring a correct binding to the many binding sites of every ligand [26,27]. The epidermal growth factor receptor (EGFR) and its downstream signaling pathways are involved in the development and progression of several human tumors. Based on the results of previous studies on the anticancer activities of phenolic compounds (e.g., eriodictyol and secoisolariciresinol) through the inhibition of the EGFR as the kinase pathway [16,28,29,30], we chose the EGFR to be the tested protein in the molecular docking study. The findings of our molecular docking study highlighted the binding activity of eriodictyol and secoisolariciresinol toward the EGFR signaling pathway, as the two promising compounds were docked inside the active site of EGFR protein with good binding energies of −19.93 and −16.63 Kcal/mol, respectively, compared with that of the co-crystallized ligand of −11.26 Kcal/mol. These values indicate the stability of compound–protein complexes (Table 4). They showed good interactions with the key amino acid Met 769 as the co-crystallized ligand. As seen in Figure 2, eriodictyol formed one hydrogen bond (HB) interaction with Met 769 through its OH group as the HB-acceptor with a bond length of 2.34 Å, while secoisolariciresinol formed one hydrogen bond (HB) interaction with Met 769 through its OH group as the HB-acceptor with a bond length of 1.77 Å.

### 2.4. Compounds (+) Eriodictyol and Secoisolariciresinol Inhibited EGFR

To validate the findings of our molecular docking studies for the good binding affinities of both (+) eriodictyol and secoisolariciresinol toward the EGFR protein, we performed EGFR kinase activity assays for each compound. As seen in Table 5, both compounds exhibited a potent EGFR inhibition of 80% at concentrations of 10 µM, with IC_50_ values of 76.16 and 80.26 nM, compared with erlotinib (IC_50_ = 78.6 nM). These results were consistent with the findings of our molecular docking studies of EGFR inhibition.

**Figure 3:**
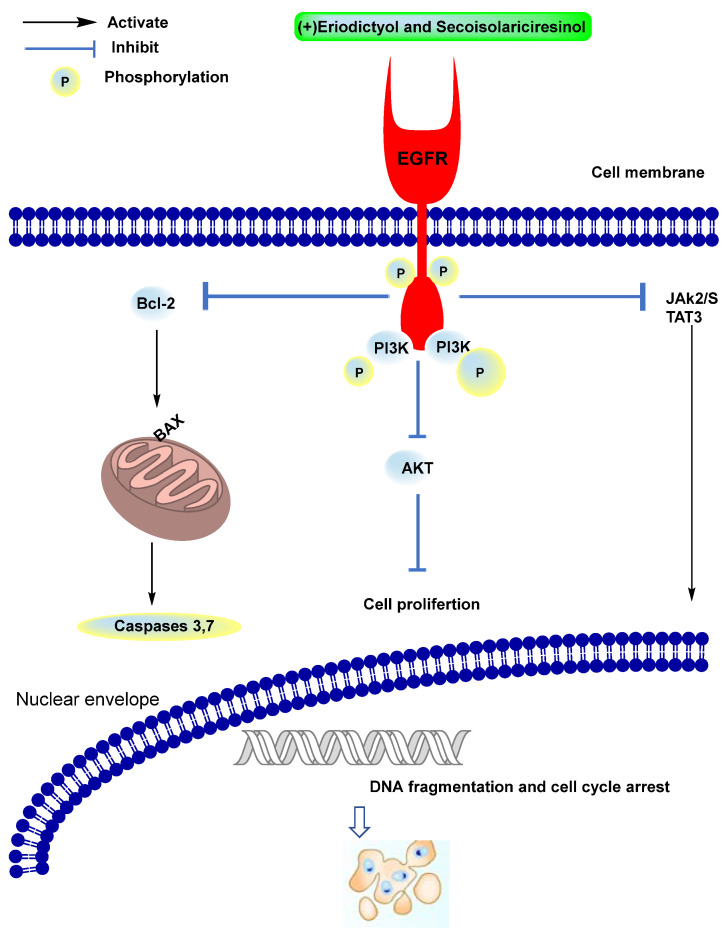
Schematic diagram of the EGFR-based pathway of anticancer activity.

As previously reported, the EGFR is involved in the pathogenesis of human carcinoma. Different mechanisms can lead to increased EGFR signaling in cancer cells: overexpression of the EGFR that, in some cases, has been associated with gene amplification; activating mutations of the EGFR; and increased expression of EGFR ligands. Targeting EGFR inhibition is a leading therapeutic objective against cancer. There are two main classes of anticancer agents affecting the EGFR: those targeting the extracellular ligand-binding domain and those blocking the intracellular tyrosine kinase (TK) domain. As shown in Figure 3, the downstream signaling pathways of EGFR, including PI3K/AKT/mTOR and JAK2/STAT3, lead to the activation of the P53 protein and other apoptosis-related genes Bax and caspase3,8,9, hence resulting in DNA fragmentation and induction of apoptotic cell death in cancer cells. Therefore, molecular mechanisms leading to intrinsic or acquired resistance to anti-EGFR drugs are common in human carcinomas [30,31,32,33].

## 3. Materials and Methods

### 3.1. Plant Material and Collection Process

*Thonningia sanguinea* was collected from the eastern region of Ghana in 2015. The plant was taxonomically identified by the aid of the specialist in the Ghana Herbarium. A voucher specimen (CSRPM no. 140) was kept in the herbarium.

### 3.2. Extraction, Fractionation, and Isolation of Pure Compounds

#### 3.2.1. Extraction Process and Fractionation Procedure

The collected *T. sanguinea* plants were air-dried in a shaded area for 7 days, then ground and soaked in methanol for 3 days. The extraction process was carried out by soaking in methanol three times (3 × 10 L), followed by soaking in a mixture of chloroform and methanol (1:1; 3 × 10 L) 3 times, to ensure complete extraction. The combined methanolic and chloroform/methanolic extracts were collected and concentrated in vacuo to produce the crude extract of *T. sanguinea* [2]. An amount of 97 g of the crude extract was dissolved in distilled water and successively fractionated by partitioning with organic solvents of different polarities to produce the *n*-hexane (7 g), ethyl acetate (43 g), *n*-butanol (32 g), and aqueous (15 g) fractions.

#### 3.2.2. Isolation of Pure Compounds

The pure compounds were isolated and structurally elucidated using ^1^H NMR and ^13^C NMR spectroscopic analysis as previously reported [2].

### 3.3. Gas Chromatography Coupled to Tandem Mass Spectrometry (GC-MS/MS)

#### 3.3.1. Calibration Graph of Isolated Compounds (**1**–**5**)

The five isolated compounds (**1**–**5**) were further analyzed using gas chromatography coupled with tandem mass spectrometry (GC-MS/MS). Equal amounts of 100 mg of each isolated compound were dissolved in 100 mL of methanol to prepare a methanolic stock solution of a mixture of different standards at a concentration of 1 mg/mL. The stock solution was kept in a refrigerator to construct a calibration curve.

#### 3.3.2. GC-MS/MS Analysis Conditioning

The GC-MS/MS analysis was performed using a gas chromatograph (Agilent Technologies 7890A, Santa Clara, CA, USA) interfaced with a mass-selective detector (MSD, Agilent 7000 Triple Quad, Santa Clara, CA, USA) equipped with an Agilent HP-5ms (5%-phenyl methyl polysiloxane) capillary column with dimensions of 30 m × 0.25 mm i.d. and 0.25 μm film thickness. Helium was used as a carrier gas with a flow rate of 1.3 mL/min. The temperature program was established as follows: the chromatographic analysis started isothermally at 55 °C for 5 min, followed by a gradual increase in temperature up to 270 °C over 10 min, and held for 5 min. The injection volume was adjusted to 1 µL, and the mass spectrometer was operated in a full scan mode for a mass range of 50–600 amu. The ionization energy was set to 70 eV, and the temperature of the ion source was 250 °C. Finally, the spectral data of the standard isolated compounds were confirmed with those found in the NIST98 and Wiley275 MS libraries.

#### 3.3.3. Plant Sample Assay

The methanolic crude extract of *T. sanguinea* and its ethyl acetate fraction were dissolved to a concentration of 1 g/10 mL, individually, then each was diluted tenfold. Both diluted solutions were applied under the above-mentioned conditions [34]. Then, the compounds under investigation were identified by comparing their retention time and spectral data with those of the standard isolated compounds.

### 3.4. Molecular Docking Studies

The virtual mechanism of binding of the identified compounds (**1**–**5**) toward the active site of the epidermal growth factor receptor, EGFR, (PDB = 1M17) was assessed. All the tested compounds were chemically and energetically optimized. Additionally, the protein structure was freely accessible from the PDB. The molecular docking study was carried out following routine preparation of the appropriate formats of receptor and ligands; determination of a grid box with dimensions of 10 Å in the x, y, and z directions centered on the ligand; and, finally, docking with binding activities in terms of binding energies and ligand-receptor interactions [35]. MOE 2019 (Molecular Operating Environment Chemical Computing Group, Montreal, QC, Canada) was used as the validated molecular docking calculation, and Chimera software was used as the visualization software for the analysis of drug–target interactions.

### 3.5. EGFR Protein Kinase (PK) Inhibition

An EGFR-TK assay kit ADP-Glo™ kinase assay (Cat No.V9261, Promega, Madison, WI, USA) was used to evaluate the inhibitory potency of compounds (+) eriodictyol and secoisolariciresinol against the EGFR. The autophosphorylation percentage inhibition by compounds was calculated using the following equation: 100 − [control/treated − control] using the curves of percentage inhibition for eight concentrations of each compound [36,37].

## 4. Conclusions

In conclusion, a novel, simple, and sensitive GC-MS/MS method was developed for the quantification of epipinoresinol, *β*-sitosterol, eriodictyol, betulinic acid, and secoisolariciresinol in the *Thonningia sanguinea* crude extract and its ethyl acetate fraction. The validation data indicated that the developed method is highly sensitive, accurate, simple, and precise. As the compounds were analyzed in the crude extract, which is well-known as a complicated mixture of components, this standardized method can be readily applied for rapid screening and quantification of these compounds in different pharmaceutical dosage forms. The findings of the molecular docking study highlighted the binding affinities of the analyzed compounds toward the EGFR by maintaining the binding disposition as the co-crystallized ligand. So, the ethyl acetate fraction of *T. sanguinea* may serve as a rich source of EGFR inhibitors that act as a line of defense against cancer. We recommend conducting further nanotechnology-based studies of the ethyl acetate fraction in the future.

## Figures and Tables

**Table 1:** Analytical parameters for *β*-sitosterol, betulinic acid, eriodictyol, epipinoresinol, and secoisolariciresinol using the proposed GC-MS/MS method.

Parameter	*β*-Sitosterol	Betulinic Acid	Eriodictyol	Epipinoresinol	Secoisolariciresinol
Concentration range (μg/mL)	7.0–12.0	7.0–12.0	7.0–12.0	7.0–12.0	7.0–12.0
Correlation coefficient (R^2^)	0.9851	0.9928	0.9773	0.9937	0.9960
LOD (μg/mL)	0.08	0.06	0.08	0.12	0.11
LOQ (μg/mL)	0.26	0.18	0.25	0.4	0.38
Slope (b)	2071.8	4704.4	996.82	1223.2	738.37
Intercept (a)	74,876	13,942	68,478	4446.2	25,573

**Table 2:** Accuracy and precision of the proposed GC-MS/MS method for quantification of *β*-sitosterol, betulinic acid, eriodictyol, epipinoresinol, and secoisolariciresinol.

Analytes	Added Conc. (μg/mL)	Intra-Day		Inter-Day	
		Recovery% ± SD	RSD	Recovery% ± SD	RSD
*β*-Sitosterol	7.0	99.21 ±1.01	1.02	99.56 ± 1.11	1.11
	10.0	100.25 ±1.21	1.21	100.35 ±1.35	1.35
	12.0	101.26 ± 0.89	0.88	99.95 ±1.45	1.45
Betulinic acid	7.0	100.67 ± 1.09	1.08	101.11 ±1.21	1.20
	10.0	98.90 ± 1.21	1.22	100.91 ± 1.12	1.11
	12.0	99.45 ± 1.45	1.46	99.15 ± 1.32	1.33
Eriodictyol	7.0	100.13 ± 1.33	1.32	99.93 ± 1.14	1.14
	10.0	99.93 ± 0.85	0.85	98.56 ± 1.15	1.17
	12.0	99.87 ± 0.77	0.77	99.96 ± 1.10	1.10
Epipinoresinol	7.0	101.00 ± 0.87	0.86	101.10 ± 0.99	0.98
	10.0	101.73 ± 0.92	0.90	102.31 ± 0.98	0.96
	12.0	99.64 ± 0.87	0.87	100.45 ± 0.87	0.87
Secoisolariciresinol	7.0	99.32 ±1.07	1.08	99.89 ± 1.08	1.08
	10.0	99.77 ±1.14	1.14	100.95 ± 0.98	0.97
	12.0	101.45 ± 1.01	1.00	102.31± 1.34	1.31

**Table 3:** Determination of *β*-sitosterol, betulinic acid, eriodictyol, epipinoresinol, and secoisolariciresinol in herbal extracts using the proposed GC-MS method.

Samples	Average Conc. (μg/mL)				
	*β*-Sitosterol	Betulinic Acid	Eriodictyol	Epipinoresinol	Secoisolariciresinol
Methanol crude extract	21.1 ± 0.56	0.88 ± 0.06	22.74 ± 0.71	11.0 ± 0.33	7.22 ± 0.21
Ethyl acetate fraction	5.5 ± 0.12	0.86 ± 0.05	38.9 ±0.89	23.8 ±0.67	24.08 ±1.02

**Table 4:** Ligand–receptor interactions of (+) eriodictyol and secoisolariciresinol toward EGFR binding site.

Compound	Binding Affinity (Kcal/mol)	Type of Interaction	Bond Length (A°)	Interaction Moiety Involved	Amino Acid
Co-crystallized ligand	−11.26	H-acceptor	1.70	-N-	Met 769
(+) Eriodictyol	−19.93	H-acceptor	2.34	-OH	Met 769
Secoisolariciresinol	−16.63	H-acceptor	1.77	-OH	Met 769

**Table 5:** EGFR-inhibition activity of the tested compounds against EGFR-PK assay.

Compound	Percentage of Autophosphorylation Inhibition at Conc. [10 µM]	IC_50_ nM *
(+) Eriodictyol	80.34 ± 2.64	76.16 ± 1.66
Secoisolariciresinol	79.62 ± 2.16	80.26 ± 1.67
Erlotinib	83.89 ± 2.03	78.65 ± 1.85

* IC_50_ values were based on mean ± SD from three independent trials. IC_50_ was calculated by non-linear regression curve fit using GraphPad prism (Dotmatics, San Diego, CA, USA).

## Data Availability

All data are available within the article.

## Supplementary Materials

The following are available online at https://www.mdpi.com/article/10.3390/molecules27134109/s1, Figure S1: GC-MS/MS chromatograms of *β*-sitosterol in a standard solution (A), in the methanolic *T. sanguinea* crude extract (B) and its ethyl acetate fraction (C); Figure S2: GC-MS/MS chromatograms of betulinic acid in a standard solution (A), in the methanolic *T. sanguinea* crude extract (B) and its ethyl acetate fraction (C); Figure S3: GC-MS/MS chromatograms of eriodictyol in a standard solution (A) in the methanolic *T. sanguinea* crude extract (B) and its ethyl acetate fraction (C); Figure S4: GC-MS/MS chromatograms of epipinoresinol in a standard solution (A), in the methanolic *T. sanguinea* crude extract (B) and its ethyl acetate fraction (C); Figure S5: GC-MS/MS chromatograms of secoisolariciresinol in a standard solution (A) in the methanolic *T. sanguinea* crude extract (B) and its ethyl acetate fraction (C).

## Sample Availability

Samples of the compounds, *β*-sitosterol, betulinic acid, (+) eriodictyol, (+) epipinoresinol, and secoisolariciresinol, are available from the authors.
