# Supplementary material for: GC-MS/MS Quantification of EGFR Inhibitors, β-Sitosterol, Betulinic Acid, (+) Eriodictyol, (+) Epipinoresinol, and Secoisolariciresinol, in Crude Extract and Ethyl Acetate Fraction of Thonningia sanguinea
Source: Molecules. 2022 Jun 26;27(13):4109. doi: 10.3390/molecules27134109 (PMC9268025; doi:10.3390/molecules27134109)
Supplement: Supplementary file 1 [file molecules-27-04109-s001.zip › molecules-1765434-supplementary.pdf]

molecules

MDPI

Article

GC-MS/MS Quantification of EGFR Inhibitors, *β*–Sitosterol, Betulinic acid, (+) Eriodictyol, (+) Epipinoresinol and Secoisolariciresinol, in Crude Extract and Ethyl Acetate Fraction of *Thonningia sanguinea*

Additional Experimental Detail

1. **Figure S1:** GC-MS/MS chromatograms of *β*-sitosterol in a standard solution (A), in the methanolic *T. sanguinea* crude extract (B) and its ethyl acetate fraction (C).
2. **Figure S2:** GC-MS/MS chromatograms of betulinic acid in a standard solution (A), in the methanolic *T. sanguinea* crude extract (B) and its ethyl acetate fraction (C).
3. **Figure S3:** GC-MS/MS chromatograms of eriodictyol in a standard solution (A), in the methanolic *T. sanguinea* crude extract (B) and its ethyl acetate fraction (C).
4. **Figure S4:** GC-MS/MS chromatograms of epipinoresinol in a standard solution (A), in the methanolic *T. sanguinea* crude extract (B) and its ethyl acetate fraction (C).
5. **Figure S5:** GC-MS/MS chromatograms of secoisolariciresinol in a standard solution (A), in the methanolic *T. sanguinea* crude extract (B) and its ethyl acetate fraction (C).

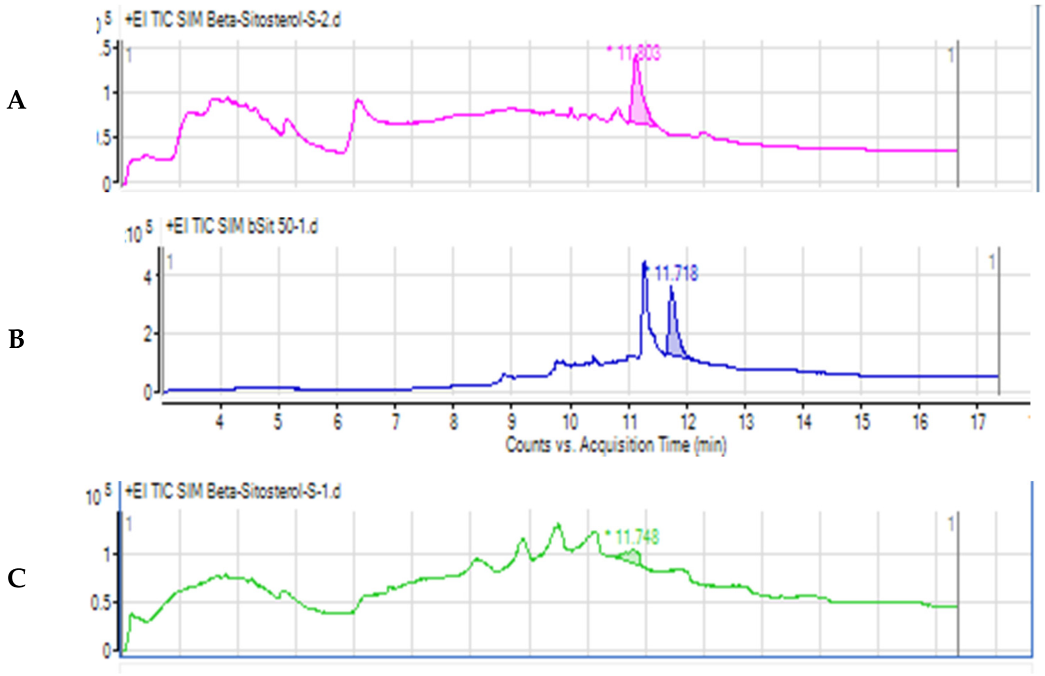

Figure S1. GC-MS/MS chromatograms of *β*-sitosterol in a standard solution (A), in the methanolic *T. sanguinea* crude extract (B) and its ethyl acetate fraction (C).

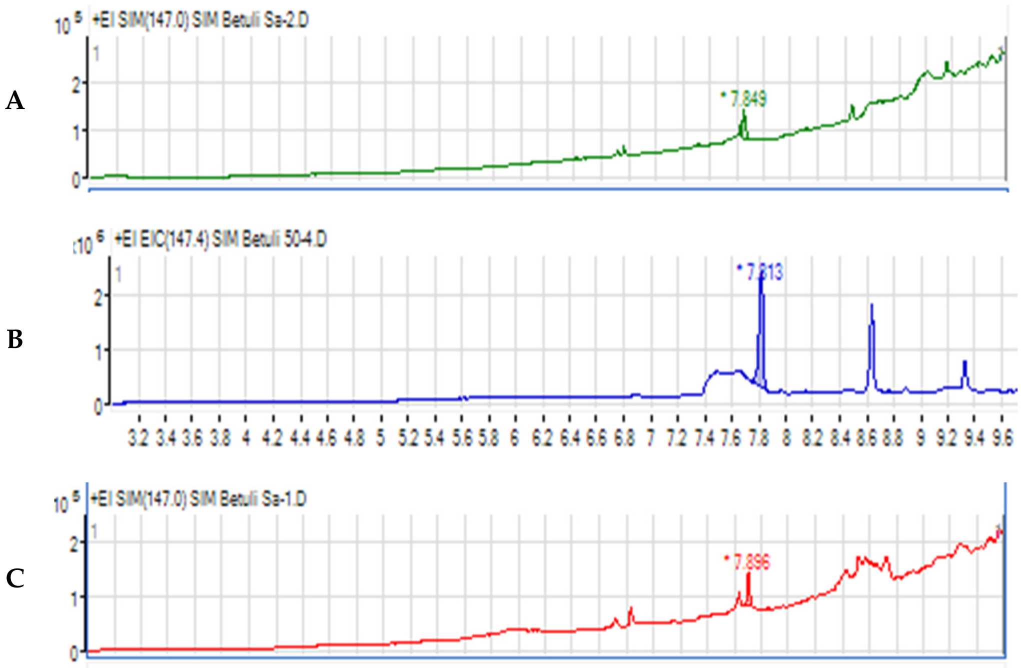

Figure S2. GC-MS/MS chromatograms of betulinic acid in a standard solution (A), in the methanolic *T. sanguinea* crude extract (B) and its ethyl acetate fraction (C).

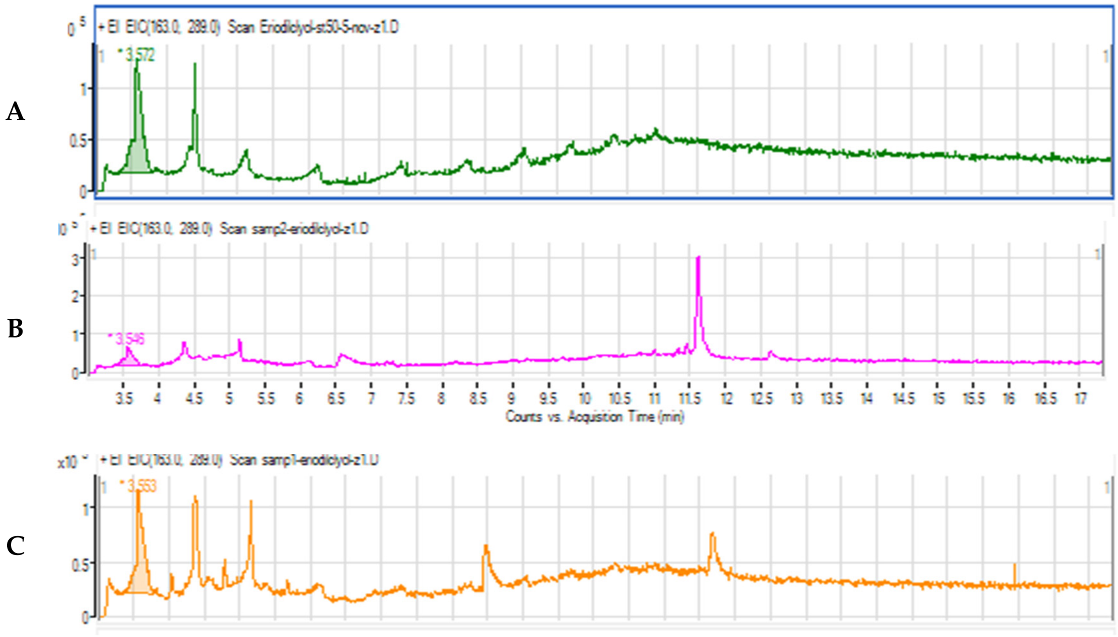

Figure S3. GC-MS/MS chromatograms of eriodictyol in a standard solution (A), in the methanolic *T. sanguinea* crude extract (B) and its ethyl acetate fraction (C).

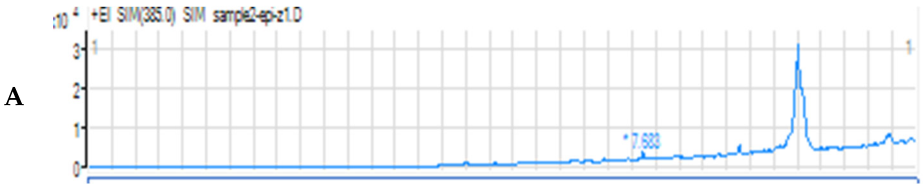

B

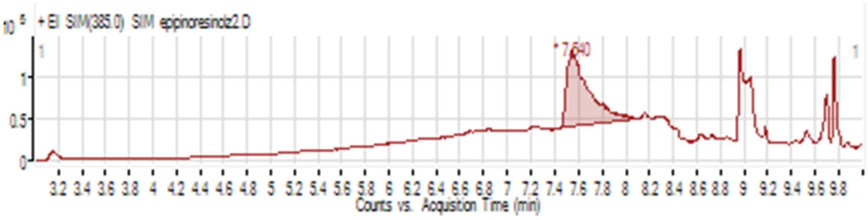

C

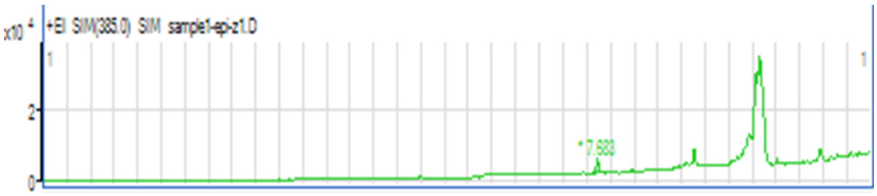

Figure S4. GC-MS/MS chromatograms of epipinoresinol in a standard solution (A), in the methanolic *T. sanguinea* crude extract (B) and its ethyl acetate fraction (C).

A

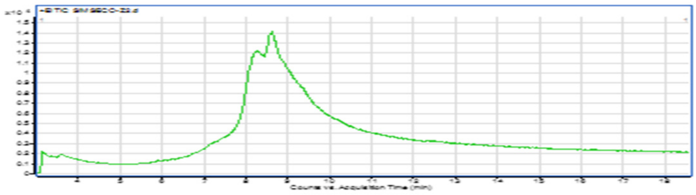

B

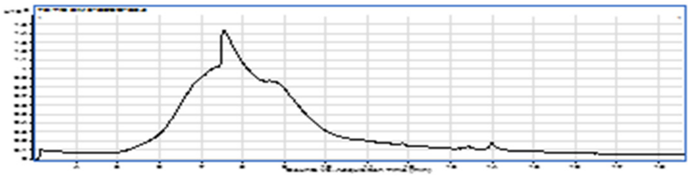

C

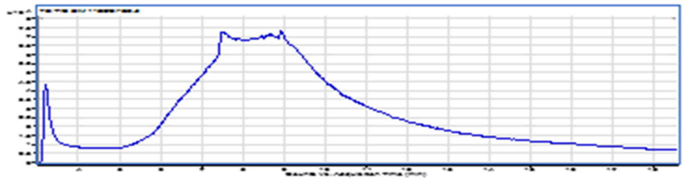

Figure S5. GC-MS/MS chromatograms of secoisolariciresinol in a standard solution (A), in the methanolic *T. sanguinea* crude extract (B) and its ethyl acetate fraction (C).
